# AnDHN, a Dehydrin Protein From *Ammopiptanthus nanus*, Mitigates the Negative Effects of Drought Stress in Plants

**DOI:** 10.3389/fpls.2021.788938

**Published:** 2021-12-24

**Authors:** Yibo Sun, Linghao Liu, Shaokun Sun, Wangzhen Han, Muhammad Irfan, Xiaojia Zhang, Li Zhang, Lijing Chen

**Affiliations:** ^1^Key Laboratory of Agricultural Biotechnology of Liaoning Province, College of Biosciences and Biotechnology, Shenyang Agricultural University, Shenyang, China; ^2^Key Laboratory of Protected Horticulture (Ministry of Education), College of Horticulture, Shenyang Agricultural University, Shenyang, China; ^3^Department of Biotechnology, Faculty of Sciences, University of Sargodha, Sargodha, Pakistan

**Keywords:** *Ammopiptanthus nanus*, drought stress, abiotic stress, legume, AnDHN, ROS

## Abstract

Dehydrins (DHNs) play crucial roles in a broad spectrum of abiotic stresses in model plants. However, the evolutionary role of DHNs has not been explored, and the function of DHN proteins is largely unknown in *Ammopiptanthus nanus* (*A. nanus*), an ancient and endangered legume species from the deserts of northwestern China. In this study, we isolated a drought-response gene (*c195333_g1_i1*) from a drought-induced RNA-seq library of *A. nanus*. Evolutionary bioinformatics showed that c195333_g1_i1 is an ortholog of *Arabidopsis* DHN, and we renamed it AnDHN. Moreover, DHN proteins may define a class of proteins that are evolutionarily conserved in all angiosperms that have experienced a contraction during the evolution of legumes. Arabidopsis plants overexpressing *AnDHN* exhibited morpho-physiological changes, such as an increased germination rate, higher relative water content (RWC), higher proline (PRO) content, increased peroxidase (POD) and catalase (CAT) activities, lower contents of malondialdehyde (MDA), H_2_O_2_ and O_2_^–^, and longer root length. Our results showed that the transgenic lines had improved drought resistance with deep root system architecture, excellent water retention, increased osmotic adjustment, and enhanced reactive oxygen species (ROS) scavenging. Furthermore, the transgenic lines also had enhanced salt and cold tolerance. Our findings demonstrate that *AnDHN* may be a good candidate gene for improving abiotic stress tolerance in crops.

**Key Message:** Using transcriptome analysis in *Ammopiptanthus nanus*, we isolated a drought-responsive gene, *AnDHN*, that plays a key role in enhancing abiotic stress tolerance in plants, with strong functional diversification in legumes.

## Introduction

For plant scientists, dramatically improving crop yields to meet the needs of an ever-increasing human population is an urgent matter at present and will become more pressing in the near future (Eshed and Lippman, 2019; Zhang et al., 2020a). Crop growth and production are strictly limited by various abiotic stress conditions that work individually or together, such as drought, salt, cold, and heat (Zhu, 2016; Wei et al., 2019). Among them, drought plays a pivotal role in affecting crop yields, greater even than the annual loss in crop yield caused by all other abiotic stresses worldwide (Zhu, 2016; Feng et al., 2020b; Gupta et al., 2020). Thus, a clearer understanding of the mechanisms underlying the control of drought resistance in different crop species is urgently needed.

Unlike animals that are mobile and can escape from adverse environmental conditions, plants have evolved a series of elaborate morpho-physiological and molecular drought tolerance mechanisms that enable them to survive drought stress (Ullah et al., 2017; Gupta et al., 2020). Many recent studies have focused on drought tolerance in plants, and the findings shed light on enhancing drought resistance (Fang and Xiong, 2014; Zhu, 2016; Liu et al., 2019; Xing et al., 2019; Bao et al., 2020; Feng et al., 2020b; Mao et al., 2020; Yang et al., 2020). There are three strategies, drought escape, drought avoidance, and drought tolerance that plants use to adapt to different degrees of water deficiency (Manavalan et al., 2009; Luo, 2010; Ullah et al., 2017; Gupta et al., 2020). Among the three strategies, drought tolerance is the most critical strategy to prevent water loss and involves a series of physiological processes: (1) stomatal closure (reducing water loss), (2) increased root density and root length (increasing absorption of water), and (3) adjusting osmotic conditions at the cellular level by promoting the production of osmolytes, such as proline (PRO) and trehalose (Xie et al., 2006; Comas et al., 2013; Zhang et al., 2020b; Zhou et al., 2020). These key physiological responses and the expression of numerous downstream responsive genes under drought stress are mainly controlled by the hormone abscisic acid (ABA; Ullah et al., 2017; Gupta et al., 2020). In addition, a recent hypothesis proposes that COST1 coordinates with drought tolerance and autophagosome formation in *Arabidopsis* (Bao et al., 2020). Despite previous hypotheses, the functions of proteins encoded by genes involved in drought resistance remain largely unknown in many diverse plant species.

Dehydrins (DHNs) are a group of environmental stress-responsive proteins that belong to Group II of the late embryogenesis abundant (LEA) protein family. Based on the several highly conserved motifs (K-, Y-, S-, and φ-segments), DHNs can be divided into five subgroups, such as YnSKn, YnKn, SKn, Kn, and KnS (Rorat, 2006). The K-segment, harboring a lysine-rich repetitive domain (EKKGIMDKIKEKLPG), is an exclusive conserved motif present in all DHNs, and this segment may play a pivotal role in protein-lipid interactions (Close, 1996; Koag et al., 2009). The rest three motifs are not essential to characterize the DHN proteins. The Y-segment, a conserved sequence [(T/V) D (E/Q) YGNP] showing partial identity to plant and bacterial chaperones binding sites, is found in the N-terminal region of DHNs (Malik et al., 2017). The S-segment, consisting of four to eight serine residues modifiable by phosphorylation, may regulate protein conformations and ion-binding activities (Yang et al., 2012). The poorly conserved regions, so-called φ-segments, are enriched with polar amino acids (Vornam et al., 2011; Graether and Boddington, 2014).

In response to abiotic stresses (e.g., drought, osmotic stress, salinity, and temperature), many *DHN* genes are upregulated in all vegetative tissues (Close, 1997; Nylander et al., 2001; Kosová et al., 2007; Peng et al., 2008; Kim and Nam, 2010; Shekhawat et al., 2011; Riyazuddin et al., 2021). In *Arabidopsis*, overexpression of EARLY RESPONSIVE TO DEHYDRATION 10 (ERD10), LOW TEMPERATURE-INDUCED 30 (LTI30), and DHN5 enhance freezing and salt tolerance (Puhakainen et al., 2004; Brini et al., 2007). *OsDHN1* has been shown to play a core role in drought and salt stress (Kumar et al., 2014; Verma et al., 2017). *ShDHN* has been reported to promote resistance against drought and cold stress (Liu et al., 2015), and *MsDHN1* can increase tolerance to Al stress in *Medicago sativa* (Lv et al., 2021). In addition to abiotic stresses, there is evidence that LEA family proteins are involved in antibacterial activity. Overexpression of *LEA2* and *LEA4* derived from *Arabidopsis* in *Escherichia coli* both leads to the inhibition of bacterial growth (Campos et al., 2006). The conserved K-segment peptides are responsible for the antibacterial activities against Gram-positive bacteria (Zhai et al., 2011). While DHN functions remain elusive in other species though a few clues have been found.

In China, *Ammopiptanthus nanus* (*A. nanus*; Leguminosae) is a rare and endangered species. *A. nanus* is an evergreen broadleaf shrub that is only found in severely arid regions of the Xinjiang Uygur Autonomous Region in northwestern China (Liu et al., 2016, 2019). The weather in this region can be extreme, with temperatures varying from −29.3°C to 34.7°C, annual precipitation of <200 mm, and annual evaporation >2,500 mm (Liu et al., 2016, 2019). Thus, *A. nanus* is an excellent species in which to study the mechanisms underlying drought resistance. Because species in the legume family provide abundant and sustainable food, feed, and industrial materials worldwide, elucidating the abiotic stress resistance mechanisms in *A. nanus* will provide a theoretical basis for enhancing abiotic stress tolerance in other legumes.

In this study, we successfully isolated and characterized a drought response gene (*c195333_g1_i1*, NCBI GenBank: AFH89648) from *A. nanus* using rapid amplification of cDNA ends (RACE). The evolutionary history of this gene family in monocots and eudicots is reconstructed, and we renamed it *AnDHN*, the exclusive *DHN* is isolated from a drought-induced RNA-seq library of *A. nanus*. Moreover, we show that the expression of *AnDHN* is induced by drought stress. Overexpression of *AnDHN* in Arabidopsis enhances drought tolerance through increased root length, excellent water retention, and enhanced ROS scavenging. Intriguingly, the promoter of *AnDHN* potentially responds to multiple abiotic stresses and hormones. *AnDHN* is further shown to act as a positive factor against salt and cold stress. Overall, our results will potentially be beneficial to agriculture by introducing genes from resilient legume crops to counter the effects of changing environmental conditions to meet the needs of a growing global human population.

## Materials and Methods

### Plant Material and Growth Conditions

*Ammopiptanthus nanus* seeds were collected from Wuqia County (39°72′N, 75°26′E), Xinjiang Uygur Autonomous Region, China. Seeds were sown in a Murashige and Skoog (MS) solid medium and germinated in an artificial growth chamber under the following conditions: 16-h light/8-h dark at 25°C. Abiotic stress was applied to 20-day-old seedlings of different periods (0, 6, 12, 18, 24, and 48 h) with 4°C, 20% PEG 8000, and 0.25 M NaCl as our previous study (Liu et al., 2019). In addition, the seedlings were treated with different concentrations of hormones (ABA, 2 μM; IAA, 1 μM; NAA, 2 μM; MeJA, 10 μM, ETH, 50 μM; and GA_3_, 50 μM), according to the previous study (Liu et al., 2019). The different tissues of roots, stem, and leaves were then frozen with liquid nitrogen and conserved at −80°C for RNA isolation.

The wild-type (WT) *Arabidopsis thaliana* (*Arabidopsis*) seeds used for transformation in this study were ecotype “Columbia” (Col-0). The seedlings were grown in a standard growth chamber (60% humidity, and 120 μE m^–2^ s^–1^ at 22°C) with a 16-h/8-h light/dark cycle.

### RNA and DNA Extraction

For real-time quantitative PCR analysis, three *A. nanus* tissues, such as roots, stems, and leave materials, were collected for total RNA extraction with a TRIzol reagent (Invitrogen, Carlsbad, CA, United States) as described by Liu et al. (2019). Moloney Murine Leukemia Virus (M-MLV) reverse transcriptase (Promega, Madison, WI, United States) was used for first-strand cDNA synthesis. The genomic DNA was isolated from the young leaf (0.4 cm length) of *A. nanus* with a Plant Genomic DNA kit (Tiangen, Beijing, China). Total RNA and DNA quality was determined using a BioDrop μLITE+ (Harvard Bioscience Shanghai).

### 5′, 3′ Rapid Amplification of cDNA Ends of *AnDHN*

Gene-specific primers for *AnDHN* were designed with primer-blast^1^ using partial sequence fragment that was obtained from transcriptome sequencing results. The partial cDNA sequence was isolated with a SMART RACE cDNA Amplification Kit (Clontech, San Jose, CA, United States). The cDNA pools for 3′ and 5′ RACE were generated with the total RNA extracted from leaves of *A. nanus*. The product of reverse transcription was used for outer PCR with the 5′ and 3′ RACE outer primers (gene-specific primers, GST1 and GST4, respectively), and the first-round products were further used for the inner PCR (gene-specific primers, GST2, GST3, and GST5). The detail sequences of outer and inner primers are described in Supplementary Table 4. The final PCR products were purified and cloned into *pGEM-T* vector (Takara, Dalian, China). The positive clones were extracted as recombinant plasmids. More than three independent positive clones were sequenced by Sangon Biotech (Shanghai, China). The full-length cDNA of *AnDHN* was obtained by aligning and assembling to the 5′ and 3′ sequences with an overlapping fragment using Vector NTI Advance^®^ 11.5 software.

### Phylogenetic Analysis

For the phylogenetic tree of DHNs, we obtained multi-species DHN protein sequences from Phytozome 13^2^ (Goodstein et al., 2012) and aligned with Clustal X version v2.1 with default parameters (Larkin et al., 2007). A maximum-likelihood (ML) phylogenetic tree was constructed with IQ-Tree v1.6 using JTT + F + G4 model as suggested by IQ-Tree model test tool (BIC criterion) with 1,000 times of bootstrap replicates, edited and visualized using iTQL^3^. The phylogenetic tree was carried out using a common taxonomy tree application of NCBI^4^, visualized using MEGA5 software (Tamura et al., 2011) and manually optimized for viewing according to recent studies (Puttick et al., 2018).

### Real-Time-qPCR

Real-time-qPCR and data analysis were conducted as described previously by Sun et al. (2020). Gene-specific primers were designed using primer-blast (see text footnote 1) with *AnACTIN* (GenBank: KJ873129.1) and *AtACTIN* as reference. Tissues from five seedlings with the same treatment were pooled for RNA extraction as one biological replicate. Three biological replicates were included for each treatment for one independent experiment, and each sample was amplified in three parallel reactions as technical replicates. PCR was carried out using Real Master Mix (SYBR Green) (Tiangen, Beijing, China) on QuantStudio 7 Flex (Applied Biosystems, Waltham, MA, United States) with 96-well format. The relative expression level was determined according to Sun et al. (2020). The 2^–ΔΔCt^ methods were used to calculate the relative expression level of *AnDHN* (Yin et al., 2020).

### Vector Construction and Subcellular Localization

The *AnDHN* coding sequence (CDS) was fused with green fluorescent protein (GFP) driven by *CaMV 35S* promoter. The recombinational fragment was inserted into the *pCAMBIA1302* vector for subcellular localization. After sequenced, the *35S: AnDHN-GFP* constructions were transformed into *Agrobacterium tumefaciens* GV3101 strain. Agrobacterium tumefaciens transformant strains were grown overnight at 28°C in 20 ml yeast extract mannitol broth (YEB) medium plus selective antibiotics, collected by centrifugation, and resuspended in infiltration medium (2 M MgCl_2_, 0.2 M MES, and 100 mM acetyleugenone, PH = 5.7) until the optical density (OD) value was 0.5–1.0. About 5-week-old *Nicotiana benthamiana* leaves were infiltrated with the bacterial cell suspensions and the plasma marker PAD62. GFP fluorescence signals were observed and documented under a confocal laser-scanning microscope (Olympus Fluoview Ver. 2.0c Viewer) after 48 h in the dark. This experiment was repeated for three biologicals, and each assay was performed with three *N. benthamiana* leaves.

### Isolation of the *AnDHN* Promoter

The *AnDHN* promoter was isolated from *A. nanus* genomic DNA using genome walking as described by Liu et al. (2019). For PCR reaction, the *AnDHN* gene-specific primers were designed according to the sequence of *AnDHN* full-length cDNA. Putative functional *cis-*acting elements (CRE) of the *AnDHN* promoter were identified by the PlantCARE database^5^, and the transcription factor-binding sites (TFBSs) were predicted by JASPAR^6^ (Wasserman and Sandelin, 2004).

### Arabidopsis Transgenic Lines Isolation

The CDS was driven by the *CaMV 35S* promoter. The recombinational fragment was inserted into the *pCAMBIA3301* vector for genetic transformation. Arabidopsis transformation was performed by the floral dip procedure (Clough and Bent, 1998). The seeds were collected from the infiltrated plants and selected on a half-strength MS medium containing 50 μg/ml of hygromycin. Hygromycin-resistant plants were transferred to soil 10 days after germination. The progeny of three lines with 3:1 segregation was further treated with hygromycin reagents to screen for the homozygous single insertion. Finally, 10 independent overexpressed T_3_ lines were obtained. Three representative T_3_ independent lines (*AnDHN#1*, *AnDHN#2*, and *AnDHN#3*) were used for subsequent scoring phenotypes.

### Abiotic Stress Tolerance Assays Statistical Analysis

For seeds germination assay, seeds of WT and *AnDHN* transgenic lines were germinated under vernalization conditions (4°C) for 3 days. Then, seeds were on a half-strength MS solid medium containing different concentrations of mannitol (0, 200, 300, and 400 mM) and NaCl (0, 100, 150, and 200 mM). To assess cold tolerance, seeds were sown in a normal half-strength MS solid medium and vernalize treatment for 2 days. Then, the samples were transferred to a low-temperature artificial growth chamber at 4°C for 4 days. And then the seeds were transferred to another growth chamber at 22°C for 5 days. Germination rates were counted for different abiotic stress for three biological replicated assays. For every biological replicate, we tested at least 30 seeds of each line from the same batch three times as one technical replicate.

For the root growth experiment, seeds of WT and *AnDHN* transgenic lines were transferred to a constant temperature incubator at 22°C for 4 days after vernalization. Then, these seeds further on a half-strength MS solid medium containing mannitol (0, 300 mM) and NaCl (0, 150 mM) were grown in a chamber with a condition (16/8 h d/night at 22°C) for 7 days. Meanwhile, another part of the seeds was also sown on a normal half-strength MS solid medium has grown in a chamber with a condition (16/8 h d/night at 4°C) for 7 days. The root length of seedings of WT and *AnDHN* transgenic lines was collected and measured. Average values were calculated from three biological replicates. And for every biological replicate, we tested six seedlings from the same batch.

Ion leakage (IL) was according to Sakuraba et al. (2014) with minor modifications. The leaves were placed in a tube with 25 ml of double-distilled water for 2 h, and then the initial conductivity (C1) was detected. After heated in boiling water for 15 min and cooled until to room temperature, the final electrolyte conductivity (C2) was detected. Hence, the relative IL (%) = C1/C2 × 100% was obtained. At least three independent biological replicates were analyzed for each line, and no less than 10 seedlings were collected as one sample for each biological replicate.

Relative water content was calculated according to the previous study described in Vysotskaya et al. (2010) with minor modifications. Fresh weight (FW) was measured, and the leaves were in double-distilled water for 4 h in darkness at room temperature. The turgid weight (TW) further was recorded. For dry weight (DW), the leaves were dried at 80°C for 24 h. Relative water content (RWC) equation, RWC (%) = [(FW–DW)/(TW–DW)] × 100%. Three biological replicates were used for each line, and at least 10 seedlings were collected as one sample detected for each biological replicate.

Malondialdehyde (MDA) content was detected using a maleic dialdehyde assay kit (A003-3, Nanjing Jiancheng Bioengineering Institute, Nanjing, China). Three biological replicates for each sample and at least 10 seedlings were collected as one sample for each biological replicate.

The ROS scavenging enzymes activities of PRO content, catalase (CAT) activity, superoxide dismutase (SOD) activity, peroxidase (POD) activity, and glutathione (GSH) content were detected with kits also produced by Nanjing Jiancheng Bioengineering Institute (Nanjing, China). Three biological replicates for each sample and at least 10 seedlings were collected as one sample as one biological replicate.

For histochemical staining assays, the leaves were collected from 2 to 3-week-old seeding of WT and *AnDHN* transgenic lines treated with mannitol (300 mM), NaCl (150 mM) and 4°C. The histochemical staining assays were conducted with 0.2% nitro-blue tetrazolium (NBT) and 1 mg/ml 3.3′-diaminobenzidine (DAB) solutions with vacuum infiltration for three times. The leaves were kept for 12 h in NBT and DAB solutions. Then, the leaves were decolorized by boiling in 75% ethanol. Images were visualized using stereomicroscopy (SZX16, Olympus).

These experiments were repeated at least three times. All of the statistical analyses were performed using IBM SPSS statistics 19. Significant variation was estimated with Student’s *t*-test, *p < 0.05; ^**^p < 0.01; ^***^p < 0.001. The data were presented as the mean ± SD of three independent experiments.

## Results

### A Drought-Responsive Gene in *A. nanus*

**FIGURE 1:**
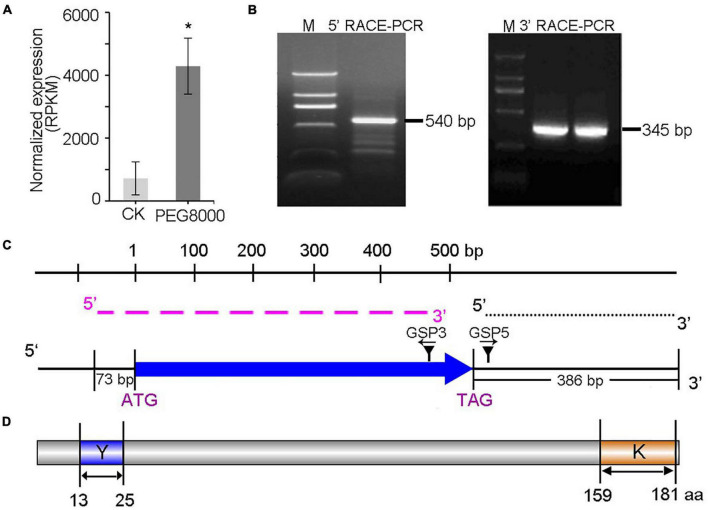
Molecular identification of a drought-responsive gene in *A. nanus.* **(A)** Expression of *A. nanus c195333_g1_i1* with PEG 8000 treatment revealed by RNA-seq analysis. RPKM value was given. Significant variation was estimated with Student’s *t*-test, *, *p* < 0.05. **(B)** The final round of 5′ RACE and 3′ RACE PCR products was generated from the leaves. The final 5′ RACE product was 540 bp and the final 3′ RACE product was 360 bp. Note that 5′ RACE product and 3′ RACE product amplified with primers from the SMARTer RACE kit and the gene special primers given in Supplementary Table 3 for detailed information. **(C)** Schematic of 5′ UTR, 3′UTR and coding sequence of *c195333_g1_i1* structure obtained by 5′ RACE and 3′ RACE. The full length for the mRNA of *c195333_g1_i1* in *A. nanus* was 1005 bp consist of a coding sequence (515 bp) marked with blue arrow, a 5′ UTR (96 bp) and 3′ UTR (360 bp). Black dotted line and rose dashed line were represent the final 5′ RACE product was 540 bp (marked with black dot line) and the final 3′ RACE product was 360 bp (marked with rose dashed line). **(D)** The protein domain structure of *c195333_g1_i1* in *A. nanus.* The conserved Y domain was shown in blue and K domain was shown in orange. RACE, rapid amplification of cDNA ends.

To identify the key regulator of drought resistance in *A. nanus*, a significantly upregulated candidate gene (*c195333_g1_i1*) (NCBI GenBank: AFH89648) was obtained from an RNA-seq library prepared from drought-induced *A. nanus* seedlings (Figure 1A and Supplementary Table 1). Lacking genomic information, the full-length sequence of this gene was isolated using 5′ and 3′ RACE. The gene-specific primers were designed from the partial sequence obtained from the RNA-seq data for degenerate RT-PCR (Figure 1C). As a result, the final 5′- and 3′-fragments were amplified through three and two rounds, respectively (Figure 1B and Supplementary Figure 1A), and the final PCR products were sequenced. Both the final 5′- and 3′- fragments (540 and 345 bp, respectively) were aligned, and the coding sequence was determined to be 543 bp in length, encoding a putative protein of 181 amino acids (Figure 1D). The final obtained 5′ UTR and 3′ UTR sequences were 73 and 386 bp, respectively. And there is no intron and only one exon exists (Figure 1C). The protein had a predicted molecular weight of 18,125.87 MW and a theoretical pI of 5.74. Using the online servers NCBI-CDD and SMART, two conserved motifs, called Y- (DEYGNPV) and K- (KKGIMNKIKEKLPGY) segments, were identified (Figure 1D), which might play key roles in AnDHN protein. These results demonstrate that *c195333_g1_i1* is a new drought-responsive gene from *A. nanus*.

### Evolution of the Dehydrin Proteins

**FIGURE 2:**
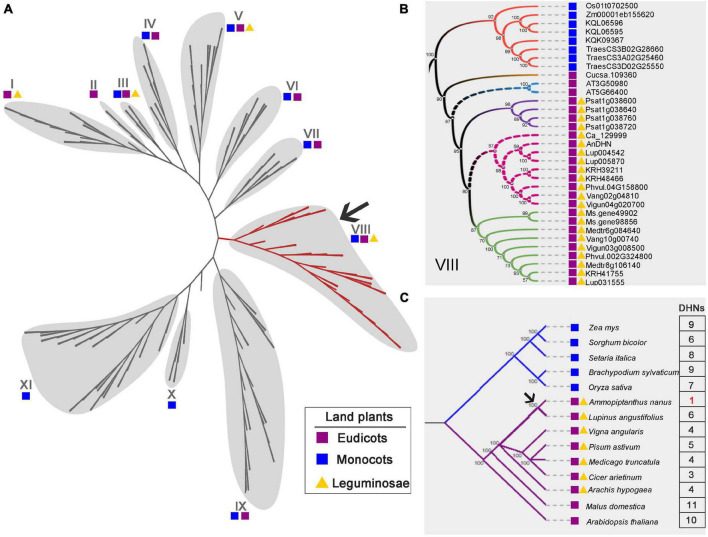
Phylogenetic relationship of DHN family members. **(A)** A maximum-likelihood phylogenetic tree of DHN proteins obtained from angiosperm, shown as an unrooted cladogram. The 11-defined DHN clusters were delineated by gray balloons. The cluster of VIII was highlighted in red and most of this cluster proteins were from legume species. The fully annotated tree was presented in Supplementary Figure 2. Each colored block represents different category species. The purple box represents monocots, namely, *Triticum aestivum*, *Brachypodium distachyon*, *Solanum tuberosum*, *Oryza sativa*, *Setaria italica*, and *Zea mays*. The blue box represents eudicots, consisting of Arabidopsis, *Malus domestica*, *Rosa chinensis*, *Solanum lycopersicum*, *Medicago truncatula*, *Glycine max*, *Cicer arietinum*, *Cucumis sativus*, *Vigna unguiculata*, *Arachis hypogaea*, *Medicago sativa*, *Pisum sativum*, *Vigna angularis*, *Phaseolus vulgaris*, and *Lupinus angustifolius*. In addition, legume species were marked by yellow-colored triangle. **(B)** Magnified view of cluster VIII. c195333_g1_i1 (AnDHN) could be the ortholog of AT3G50980 and AT5G66400, two DHN from Arabidopsis. AnDHN was closely related to Lup004542 and Ca_12999, which were belong to *L. angustifolius* and *C. arietinum*, respectively. **(C)** A simplified model showing of *AnDHN* genes occurred posterior to the split of *A. nanus* from some special eudicots, monocots, and legumes species (pointed by an arrow). Numbers indicated *DHN* genes number in different species. The different colors are only used to distinguish between different phylogenetic clades. The number in red is used only to highlight the number of genes in *A. nanus*.

Because of its adaptation to extreme environmental conditions, *A. nanus* is an excellent model species in which to study the drought and cold tolerance in legumes and other species. To further understand the evolutionary history of c195333_g1_i1, we constructed a phylogenetic tree. The ML phylogenetic tree was derived from an alignment of 189 DHN sequences from 23 species of both monocots and eudicots (Figure 2A and Supplementary Figure 2). Based on our results, the gene from *A. nanus* encoding a protein could be orthologous of the proteins encoded by AT3G50980 (XERO1) and AT5G66400 (RAB18) (Figures 2A,B and Supplementary Figure 2). XERO1 and RAB18 are known as DHN proteins from *Arabidopsis*. Among them, RAB18 is the protein extensively studied, which is widely reported involved in cold tolerance and drought tolerance of plants (Lång and Palva, 1992; Zou et al., 2021).

Previous studies have focused only on the structural types of DHN proteins, with little consideration given to the evolutionary relationships between the DHN family members. Our results show that the DHN family is widely distributed in angiosperms (Figure 2A). Among these, all of the DHN family members from legume species were clustered in subfamilies I, III, V, and VIII, with most found in subfamily VIII (Figures 2A,B). We identified AnDHN in *A. nanus* as being a protein closely related to Lup004542 and Ca_12999 in legumes (Figure 2B). A multiple sequence alignment showed that AnDHN harbors conserved K- and Y-segment domains similar to AT3G50980, AT5G66400, Lup004542, and Ca_12999 (Supplementary Figure 3), consistent with our bioinformatic prediction (Figure 1D). The sequences of predicted DHN proteins from *Zea mays*, *Sorghum bicolor*, *Setaria italica*, *Brachypodium sylvaticum*, *Oryza sativa*, *A. nanus*, *Lupinus angustifolius*, *Vigna angularis*, *Pisum sativum*, *Medicago truncatula*, *Cicer arietinum*, *Arachis hypogaea*, *Malus domestica*, and *Arabidopsis* were further used in phylogenetic reconstruction (Figure 2C). Interestingly, all of the monocot species had more than six DHN proteins, and the genomes of the eudicot species *Arabidopsis* and apple contain at least 10 DHN proteins of different coding for each. However, the number of *DHN* genes in legume species ranged from three to six, except for *A. nanus*, which lacks genomic information. Our findings suggest that DHNs are evolutionarily conserved in multiple species, which is consistent with previously reported (Close, 1997; Svensson et al., 2002; Rorat et al., 2006).

### Expression Pattern of AnDHN in Response to Drought Stress and Subcellular Localization of AnDHN

**FIGURE 3:**
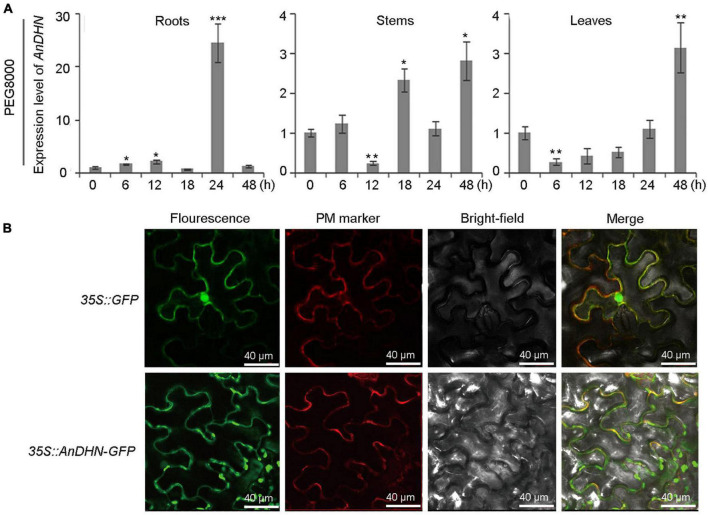
The relative expression level of *AnDHN* in different tissues and subcellular localization of AnDHN. DHN, dehydrins. **(A)** Quantitative RT-qPCR analysis revealed *AnDHN* expression level in roots, stems, and leaves at different times with PEG 8000 treatment. *AnACTIN* was used as an internal control. *AnDHN* expression was compared with that in 0 h, the value of which was set as 1. Significant variation was estimated with Student’s *t*-test, *p < 0.05, **p < 0.01, ***p < 0.001. **(B)** Subcellular localization of AnDHN-GFP in tobacco hypo epidermal cells. The green GFP fusion fluorescence overlapped with the red fluorescence of the PM marker (PAD62). Scale bars were 40 μm. DHN, dehydrins.

We next conducted a comprehensive set of experiments to functionally analyze AnDHN for its potential roles in simulated drought stress. The temporal expression patterns of *AnDHN* were analyzed in roots, stems, and leaves from 0 to 48 h of PEG 8000 treatment. Our results further confirmed that *AnDHN* is a drought-responsive gene (Figure 3A). Our data show that *AnDHN* expression in roots was significantly upregulated after 6 h of drought treatment and that expression peaked at 24 h of PEG 8000 treatment. Unlike roots, *AnDHN* expression was increased by 2.8-fold and 3.1-fold at 48 h compared to the 0 h control treatment in stems and leaves, respectively (Figure 3A). To determine why *AnDHN* expression responds to drought stress, we obtained the *AnDHN* promoter region, which was 830 bp in length, by genomic walking (Supplementary Table 2). Several types of drought-induced *cis*-acting elements were identified from the PlantCARE and JASPAR databases as expected (Supplementary Figure 4 and Supplementary Table 3). Unexpectedly, we also identified numerous cold and salt-induced elements in the promoter of *AnDHN* (Supplementary Figure 4 and Supplementary Table 3). To test our predictions, the expression pattern of *AnDHN* was analyzed after following cold and salt stress treatments. *AnDHN* expression was markedly upregulated at 12 h in the roots, 24 h in stems, and within 24 h in leaves exposed to salt stress (Supplementary Figure 5). In response to cold (4°C) treatments, *AnDHN* expression was dramatically different from the 0-h control at most times during the 48-h experiment in the roots, stems, and leaves (Supplementary Figure 5).

To determine the subcellular localization of the AnDHN protein, the AnDHN-GFP fusion was transiently expressed in *N. benthamiana* leaf epidermal cells. The result showed that GFP fluorescence overlaps with the plasma membrane (PM) marker, indicating the AnDHN protein is localized at the PM (Figure 3B).

### Overexpression of *AnDHN* Enhanced Drought Tolerance by Increased Germination Rate and Deep Root System Architecture

**FIGURE 4:**
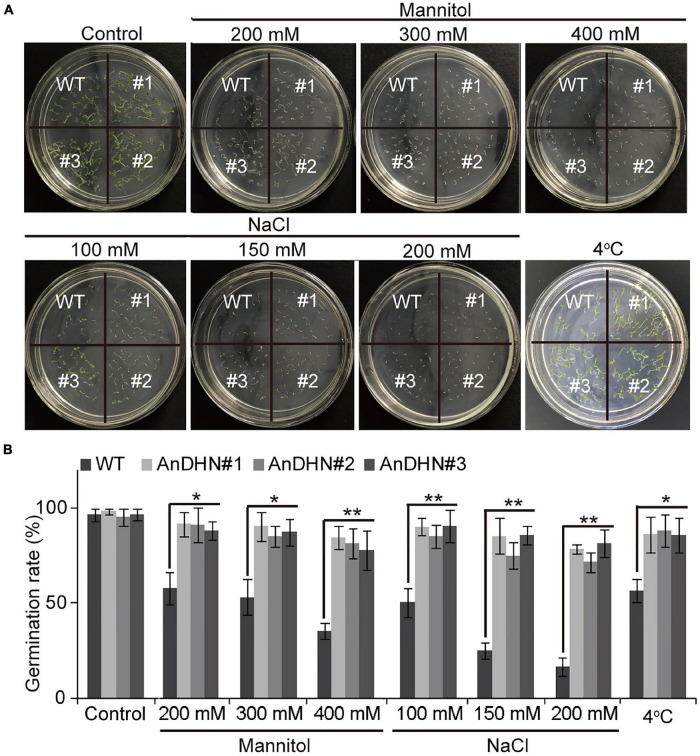
The germination rate of *AnDHN* overexpression transgenic lines. **(A)** Germination rate of the WT seedling and *AnDHN*-OX seedlings on half-strength MS medium treatment with or without 200, 300, and 400 mM mannitol; 100, 150, and 200 mM NaCl; 4°C. The average was calculated for three biological replicates and each line of seeds was pooled from more than 30. **(B)** The germination rate was calculated by ANOVA. Values were means ± SD. Significant differences based on Student’s *t*-test *p < 0.05, **p < 0.01. DHN, dehydrins.

To further verify whether *AnDHN* is related to drought stress, *AnDHN* was driven by the 35S promoter, and 10 overexpressions of *AnDHN* transgenic lines were generated in *Arabidopsis*. Among these lines, we chose three representative independent lines for further analysis (abbreviated as *AnDHN#1*, *AnDHN#2*, and *AnDHN#3*), and *AnDHN* had a relatively high expression level in these lines (Supplementary Figure 6). Germination rates in the transgenic plants showed no differences from the WT plants under normal conditions (Figures 4A,B). However, we observed higher germination rates in seeds from transgenic lines compared to seeds from WT plants at three different concentrations of mannitol (Figures 4A,B). As expected, the germination rate of WT seeds was decreased in the presence of mannitol compared to that of WT seeds without mannitol (Figures 4A,B). This may indicate that the germination rate was related to drought stress.

**FIGURE 5:**
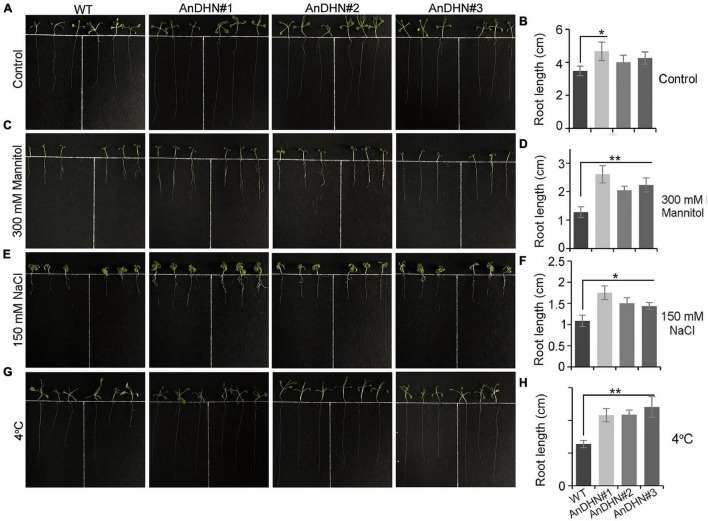
Root growth of *AnDHN* overexpression transgenic lines. **(A,C,E,G)** 4-day old seedlings of the WT and *AnDHN*-OX lines were transferred to strength MS medium with the following conditions: 1/2MS + 300 mM mannitol, 1/2MS + 150 mM NaCl, and 1/2MS + 4°C media, respectively. The average was calculated for three biological replicates. **(B,D,F,H)** The root length was calculated by ANOVA. Values were means ± SD. Significant differences based on Student’s *t*-test *p < 0.05, **p < 0.01. DHN, dehydrins.

Under drought stress, a deep root system architecture is beneficial and allows plants to adapt to adverse living conditions (Wei et al., 2020; Yang et al., 2020; Zhou et al., 2020). To confirm this, we compared the root length of WT and overexpressed plants under normal conditions and 300 mM mannitol treatment. As the result, an insignificant difference can be visualized between WT plants and overexpressing plants grown under normal conditions except for *AnDHN#1*, and there is a significant difference between the WT plants and *AnDHN#1* (Figures 5A,B). However, we found that all three transgenic lines displayed markedly increased root length when grown on a medium containing 300 mM mannitol, especially for *AnDHN#1* (Figures 5C,D). Thus, our data suggest that overexpression of *AnDHN* enhances drought tolerance by increasing the germination rate and the deep root system architecture.

### *AnDHN-OX* Plants Enhanced Drought Resistance With Excellent Water Retention, Increased Osmotic Adjustment, and Enhanced Reactive Oxygen Species Scavenging

**FIGURE 6:**
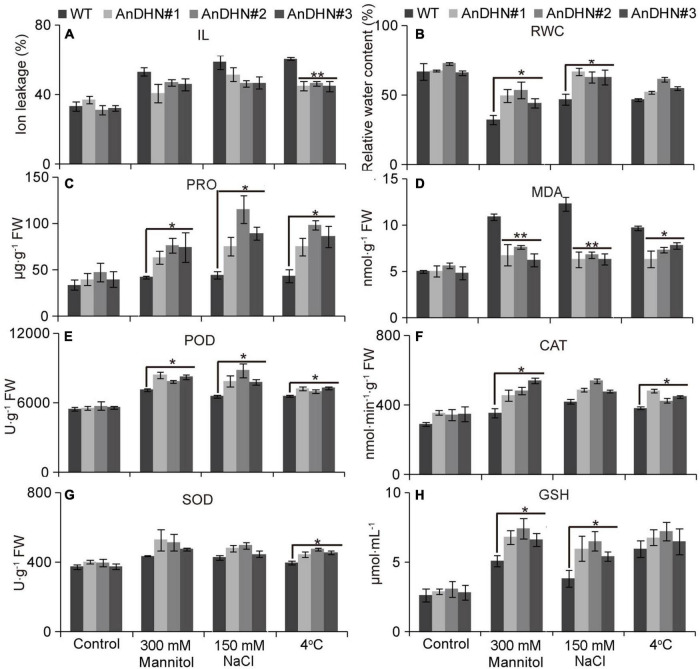
Assessment of drought, salt, and cold tolerance in WT and *AnDHN* transgenic lines. **(A)** Ion leakage (IL), **(B)** relative water content (RWC), **(C)** proline, **(D)** malondialdehyde (MDA), **(E)** peroxidase (POD), **(F)** catalase (CAT), **(G)** superoxide dismutase (SOD), and **(H)** glutathione (GSH). The WT and *AnDHN* transgenic seedlings were exposed to 300 mM mannitol, 150 mM NaCl, and 4°C on strength MS medium, respectively, and strength MS medium acted as a control. Each indicator was calculated for three biological replicates. Data were means ± SD. * and ** represented significant differences between control and *AnDHN* transgenic lines with *p* < 0.05 and 0.01, respectively. DHN, dehydrins; WT, wild-type.

Under drought stress, plants that are capable of physiological change will be able to adapt to changeable environments. We therefore investigated two physiological indexes, IL and relative water content (RWC), to investigate water retention in the plants. We found a slight decline in electrical conductivity under drought stress (Figure 6A). However, all three independent *AnDHN-OX* transgenic lines retained much more water than did the WT plants under drought stress (Figure 6B). Our results suggest that overexpression of *AnDHN* in *Arabidopsis* can enhance drought tolerance by increasing water retention.

Proline is a proteinogenic amino acid that contributes to osmotic adjustment in drought tolerance. Our assays confirmed that, when treated with 300 mM mannitol, proline accumulated to higher levels in the *AnDHN-OX* transgenic plants than in WT plants, indicating that *AnDHN-OX* plants have increased drought tolerance (Figure 6C). We next measured the MDA contents, because MDA is an important indicator of damage of cellular membranes (lipid peroxidation) caused by abiotic stresses. The MDA contents in *AnDHN-OX* lines were significantly lower than in WT plants under drought treatment (Figure 6D) showing that increased expression of *AnDHN* relieves damage to the cellular membranes.

**FIGURE 7:**
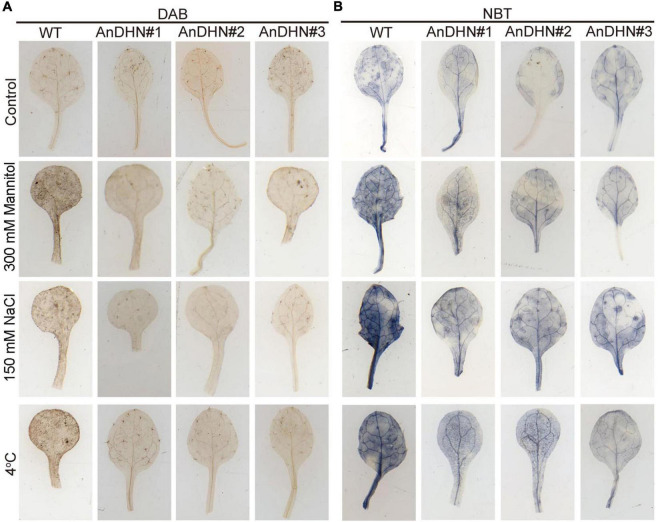
Reactive oxygen species (ROS) accumulation in WT and *AnDHN* transgenic lines under drought, salt, and cold stress. **(A,B)** DAB and NBT staining for detecting H_2_O_2_ and O_2_^–^ levels in leaves of WT and *AnDHN* transgenic lines treatment with 300 mM mannitol, 150 mM NaCl, and 4°C, respectively. Experiments are repeated three times with similar results and one representative result was shown. DHN, dehydrins.

Excess ROS (H_2_O_2_ and O_2_^–^) production can damage cellular membranes (Feng et al., 2020a), and the ROS levels are strictly controlled by antioxidant enzymes. Therefore, were also determined the activities of POD, CAT, SOD, and GSH that are responsible for ROS scavenging. As shown in Figures 6E–H, the activities of these indicator enzymes, except for SOD, were increased in the transgenic lines in response to drought stress (Figures 6E–H). Nitro blue tetrazolium (NBT) and DAB staining were performed to detect H_2_O_2_ and O_2_^–^, respectively. We observed only slight staining in the leaves of the WT and *AnDHN-OX* plants grown under normal conditions, with no visible differences between them (Figures 7A,B). Under drought stress, however, weaker staining was found in the leaves of the *AnDHN-OX* lines compared with the WT plants (Figure 7). These results indicate that *AnDHN-OX* plants had increased ROS scavenging capability in response to drought stress.

### *AnDHN* Simultaneously Enhanced Plant Tolerance to Salt and Cold Stress

In our previous study, we found that *AnDHN* expression could also be induced by both salt and cold stress (Supplementary Figure 5). Moreover, the promoter of *AnDHN* contains salt and cold stress-induced *cis*-acting elements (Supplementary Figure 4 and Supplementary Table 3). We next performed experiments to determine whether *AnDHN* affects salt and cold stress by assaying seed germination rate, root length, IL content, RWC, and the activities of crucial antioxidant enzymes. As expected, our results indeed confirmed our hypothesis that *AnDHN* simultaneously improves plant tolerance to both salt and cold stress. The *AnDHN-OX* lines had relatively higher germination rates and longer root lengths in comparison to the WT under salt and cold stress conditions (Figures 4, 5). Furthermore, the RWC and proline contents and POD and GSH activities were significantly higher in *AnDHN-OX* line plants under salt stress (Figures 6B,C,E–H). The concentrations of IL, proline, and the POD, CAT, and SOD activities in the *AnDHN-OX* lines were higher than in the controls in response to cold stress (Figures 6A,C,E–G). In addition, there was less DAB and NBT staining in the overexpression lines relative to that in the WT plants (Figure 7). These results show that *AnDHN* also has the potential to enhance salt and cold tolerance in plants.

## Discussion

### *AnDHN* Improves Drought Tolerance via Abscisic Acid

Although DHNs are predicted to be stress-related proteins (Nylander et al., 2001; Kosová et al., 2007; Peng et al., 2008; Kim and Nam, 2010; Shekhawat et al., 2011), the study of DHN functions in many species is still in its infancy. Previous studies have been focused on analyses of the putative promoters of *DHN* genes (Zhu et al., 2014; Liu et al., 2015; Aguayo et al., 2016). All of these studies showed that the expression of *DHN* genes is upregulated in response to multiple hormones and abiotic stresses (Zhu et al., 2014; Liu et al., 2015; Aguayo et al., 2016). Our results show that *AnDHN* positively improves drought tolerance through morphological and physiological changes in *A. nanus*.

Abscisic acid can improve stress tolerance in plants by allowing them to adapt to changeable environments (Zhu, 2016). Previous studies have shown that ABA regulates the expression of many downstream genes in response to drought stress with morphological and physiological changes, such as deep root system architecture, stomatal closure, and enhanced ROS scavenging (Ullah et al., 2017; Xing et al., 2019; Gupta et al., 2020). Also, ABA-dependent signaling pathways play a critical role in the response to drought stress (Ullah et al., 2017). Our data demonstrate that AnDHN is a membrane protein (Figure 3B), suggesting that *AnDHN* could be an upstream gene involved in drought tolerance. Moreover, previous studies in rice showed that overexpression of *OsDhn-Rab16D* modulated the expression patterns of ABA signaling genes to alter endogenous ABA concentrations (Tiwari et al., 2019). Thus, we can also hypothesize that *AnDHN* indirectly changes the expression of ABA biosynthesis genes or ABA signaling genes to alter physiological responses and the expression of numerous downstream tolerance genes under drought stress in *A. nanus*. To test this hypothesis, we also detected the expression of ABA biosynthetic and catabolic genes in the WT plants and *AnDHN* overexpressed lines. The results showed that the expression levels of *AtNECD3* and *AtNECD5* (two ABA biosynthetic genes) were significantly upregulated, while the expression levels of *AtCYP707A1* and *AtCYP707A3* (two ABA catabolic genes) were downregulated (Supplementary Figure 7). Interestingly, our results also show that *AnDHN* expression is induced by ABA (Supplementary Figures 4, 8 and Supplementary Table 3), indicating that there might be an AnDHN-ABA loop to improve drought tolerance in *A. nanus*.

### Subcellular Localization of Dehydrin Proteins

Previous studies have shown that DHN proteins are localized in the cytosol, nucleus, mitochondria, vacuole, and vicinity of the PM (Houde et al., 1995; Danyluk et al., 1998; Rorat et al., 2004). The localization of the DHNs in cells may depend on the existence or deficiency of the Y-, S-, and K- segments (Graether and Boddington, 2014). YnKn, YSK, and Kn types of DHN proteins have been found to localize to the nucleus and cytoplasm (Wisniewski et al., 1999; Lin et al., 2012). SKn type of DHNs is found to localize in the vicinity of the PM (Danyluk et al., 1998; Hara et al., 2003). Y-segment has shown no connection with the localization of DHN proteins. In our research, we found that the AnDHN protein was localized at the PM (Figure 3B). According to the role in this study and the previous reports (Hara et al., 2003; Yang et al., 2014), we guessed that it could play a key role in protecting lipid membranes against peroxidation under drought and freezing stress.

### Dehydrins Are Evolutionarily Conserved in the Abiotic Stress Response in Angiosperms

Dehydrin proteins are reported to be involved in responses to various abiotic stresses in plants (Hundertmark and Hincha, 2008; Xu et al., 2014; Zhu et al., 2014). In our study, we also showed that overexpression of *AnDHN* in Arabidopsis mediates the effects of salt and cold stress (Figures 4–7 and Supplementary Figure 5), in addition to its effect on drought stress. Some hormones play critical roles in abiotic stress tolerance, and various genes that are involved in regulating abiotic stress tolerance are induced by multiple hormones (Zhu et al., 2014; Liu et al., 2019; Xing et al., 2019). Changes in *AnDHN* expression following treatment with ABA, Indole-3-Acetic acid (IAA), 1-Naphthyl acetic acid (NAA), Jasmine (keto) acid methyl ester (MeJA), ethylene (ETH), and GA_3_ are shown in Supplementary Figure 8, consistent with previous results in *A. nanus*, *Cucumis melo* var. *makuwa Makino*, and *Triticum aestivum* (Zhu et al., 2014; Liu et al., 2019; Xing et al., 2019). This suggests that *AnDHN* may play an active role in abiotic stress responses in *A. nanus*. *Cis*-acting elements in promoters that affect gene expression in response to abiotic stress have been widely studied (Yamaguchi-Shinozaki and Shinozaki, 2005; Zhu et al., 2014; Liu et al., 2019). We identified many hormone-inducible elements in the *AnDHN* promoter that respond to MeJA, IAA, and ABA in addition to drought, cold, and salt (Supplementary Figure 4 and Supplementary Table 3). Moreover, the promoter harbored a lot of TFBSs of abiotic stress and hormones, simultaneously (Supplementary Table 3). Thus, our results imply that *AnDHN* expression depends upon the presence of these promoter elements to respond to abiotic stresses.

To the best of our knowledge, the DHN family has multiple members in most species; for example, there are four and 10 *DHN* genes in *Eucalyptus globulus* and Arabidopsis, respectively (Fernández et al., 2012; Aguayo et al., 2016). Phylogenetic analysis of DHN proteins from dicots and monocots showed that there are at least three members in each of the species examined, although only a single gene was identified in *A. nanus* (Figure 2 and Supplementary Figure 2). Furthermore, *AnDHN* was isolated from a drought-induced RNA-seq library of *A. nanus*. Hence, we speculate that there may be more than one *DHN* gene in *A. nanus*, which would be consistent with other species. DHN acts as the pivotal regulator in the response to various abiotic stresses in *Eriobotrya japonica*, *Eucalyptus globulus*, *Oryza sativa*, and *Arabidopsis* (Fernández et al., 2012; Kumar et al., 2014; Xu et al., 2014; Aguayo et al., 2016). We further demonstrated that AnDHN positively regulates drought stress tolerance and tolerance to salt and cold (Figures 3–7). Phylogenetic analysis and sequence alignment revealed that DHN proteins are highly conserved in angiosperms (Figure 2 and Supplementary Figure 3). Taken together, these results indicate that DHNs appear to define a class of proteins that regulate abiotic stress tolerance in all angiosperms.

Apart from the function of stress, LEA family proteins may also play key roles in the growth and development of plants. Unfortunately, research on LEA function is limited by their proteins with structural flexibility and lacking similarity with other known proteins. It will be a hotpot to explore the association between their structural types with different levels of water deficiency based on the previous studies of LEA proteins (Battaglia and Covarrubias, 2013). Moreover, numerous *LEAs* are detected in developmental root hairs, suggesting that they have the potential for water absorption and nitrogen fixation in symbiotic association rhizobium-legumes (Battaglia and Covarrubias, 2013). The research on the function of LEA proteins is not enough except for the stress response, and it will be worthwhile to investigate in the future.

### Further Perspectives of Drought Stress

*Ammopiptanthus nanus* is a non-model plant that grows in arid desert habitats in central Asia. Plants can experience conditions, such as very low rainfall, extremely high evaporation, and abnormally high and low temperatures (Liu et al., 2016, 2019). Because it is able to survive in such extreme environments, *A. nanus* has great research potential for elucidating drought and cold tolerance mechanisms that could be applied to other species. Our findings shed light on the control of drought resistance by DHN in *A. nanus*. However, the mechanisms underlying drought resistance are still largely unknown in *A. nanus* and also in other legume species.

In the future, reduced crop yields and quality loss due to drought stress will be more serious (Ullah et al., 2017). Hence the drought tolerance mechanism remaining needs to gain better exploitation. (1) From both model and non-model species, more genes responsible for drought tolerance need to be identified and characterized by genetic variation, transcriptomic, gene editing, and so on. Abiotic stress is a complicated trait. As drought often couples with others stress, pleiotropic genes are excellent. (2) Stomatal closure often with carbohydrate synthesis is reduced, how to coordinate with growth and drought stress is important. (3) Research studies of plant responses to drought are mostly studied from plants grown in the laboratory greenhouse, not natural. We need to accelerate the application of our results into natural water-deficit conditions for agriculture.

## Data Availability Statement

The original contributions presented in the study are included in the article/Supplementary Material, further inquiries can be directed to the corresponding authors.

## Author Contributions

LC conceptualized the project, had overall responsibility for this project, such as project ideas, guidance on experimental design, data analysis, manuscript writing, and revision, and took part in the project administration and funding acquisition. YS, LL, and SS carried out the laboratory work and data analysis. YS wrote the first draft of the manuscript with the help of XZ. MI helped to review and edit the manuscript. LZ has been involved in critically revising the manuscript for important intellectual content. All authors have read and approved the final manuscript.

## Conflict of Interest

The authors declare that the research was conducted in the absence of any commercial or financial relationships that could be construed as a potential conflict of interest.

## Publisher’s Note

All claims expressed in this article are solely those of the authors and do not necessarily represent those of their affiliated organizations, or those of the publisher, the editors and the reviewers. Any product that may be evaluated in this article, or claim that may be made by its manufacturer, is not guaranteed or endorsed by the publisher.

## Footnotes

1
https://www.ncbi.nlm.nih.gov/tools/primer-blast

2
https://phytozome-next.jgi.doe.gov/

3
https://itol.embl.de/

4
https://www.ncbi.nlm.nih.gov/Taxonomy/CommonTree/wwwcmt.cgi

5
http://bioinformatics.psb.ugent.be/webtools/plantcare/html/

6
http://jaspar2016.genereg.net/

## Supplementary Material

Supplementary Figure 1 The transcript of *AnDHN*. DHN, dehydrins. **(A)** Detection of the 5′ and 3′-terminus of the transcript of *AnDHN* by 5′ and 3′-RACE. The gene special primers were given in Supplementary Table 4 for detailed information. **(B)** The complete transcript of *c195333_g1_i1* was obtained by 5′ and 3′ RACE. The start and stop codons were labeled in red. DHN, dehydrins*;* RACE, rapid amplification of cDNA ends. The bold sequence represents the coding sequence of *AnDHN*.

Supplementary Figure 2 A maximum-likelihood phylogenetic tree of DHNs. The generated dataset consists of 23 species and 189 DHN proteins. DHN, dehydrins.

Supplementary Figure 3 Amino acid sequence alignments with closely related DHNs. DHNs from *A. nanus* and closely related species, namely, *Arabidopsis*, *C. arietinum*, *L. angustifolius*, *G. max*, *P. vulgaris*, *V. angularis*, and *V. unguiculata*. The Y-domain and K-domain were underlined in blue and brown lines, respectively. DHN, dehydrins. The black and gray highlights represent the conservative interval of the protein of *AnDHN*. The black highlight represents the sequence identified more than 75% and the gray highlight represents the sequence identified more than 50%.

Supplementary Figure 4 Predicted *cis-*regulatory-elements (CREs) in the 830 bp promoter regions of *AnDHN.* DHN, dehydrins.

Supplementary Figure 5 **(A–C)** Quantitative RT-qPCR analysis revealed *AnDHN* expression level in roots, stems and leaves in different times under150 mM NaCl treatments. **(D–F)** Quantitative RT-qPCR analysis revealed *AnDHN*expression level in roots, stems and leaves in different times under 4°Ctreatments. *AnACTIN* was used as an internal control. *AnDHN* expression wascompared with that in 0 h, the value of which was set as 1. Significant variationwas estimated with Student fs *t*-test *, *p* < 0.05, **, *p* < 0.01, ***, *p* < 0.001.

Supplementary Figure 6 Expression of *AnDHN* in the overexpression lines. RNA was isolated from 10-day-old seedlings. *AtACTIN* gene was used as an internal control. Significant variation was estimated with Student’s *t*-test of three independent biological replicates. ***, *p* < 0.001.

Supplementary Figure 7 The relative expression level of ABA biosynthetic and catabolic genes of WT and AnDHN overexpression lines. RNA was isolated from 10-day-old seedlings. *AtACTIN* gene was used as an internal control. Significant variation was estimated with the Student’s *t*-test of three independent biological replicates. **p* < 0.05, ***p* < 0.01, ****p* < 0.001. WT, wild-type; DHN, dehydrins.

Supplementary Figure 8 Quantitative RT-qPCR analysis revealed *AnDHN* expression level in leaves in different times. **(A–F)** Were used ABA, NAA, IAA, MeJA, ETH, GA_3_ treatment, respectively. *AnACTIN* was used as an internal control. *AnDHN* expression was compared with that in 0 h, the value of which was set as 1. Significant variation was estimated with Student’s *t*-test, *, *p* < 0.05, **, *p* < 0.01, ***, *p* < 0.001.

Supplementary Table 1 Expression level of *c195333_g1_i1* in *A. nanus* under drought treatment (RPKM).

Supplementary Table 2 List of the TFBSs and *cis-*acting elements in the 830 bp promoter of *AnDHN*. TFBS, transcription factor-binding site; DHN, dehydrins.

Supplementary Table 3 The 830 bp promoter sequence of *AnDHN.* DHN, dehydrins.

Supplementary Table 4 Primers were used in this study.
