# Supplementary figures and images for: AnDHN, a Dehydrin Protein From Ammopiptanthus nanus, Mitigates the Negative Effects of Drought Stress in Plants
Source: Front Plant Sci. 2021 Dec 24;12:788938. doi: 10.3389/fpls.2021.788938 (PMC8739915; doi:10.3389/fpls.2021.788938)

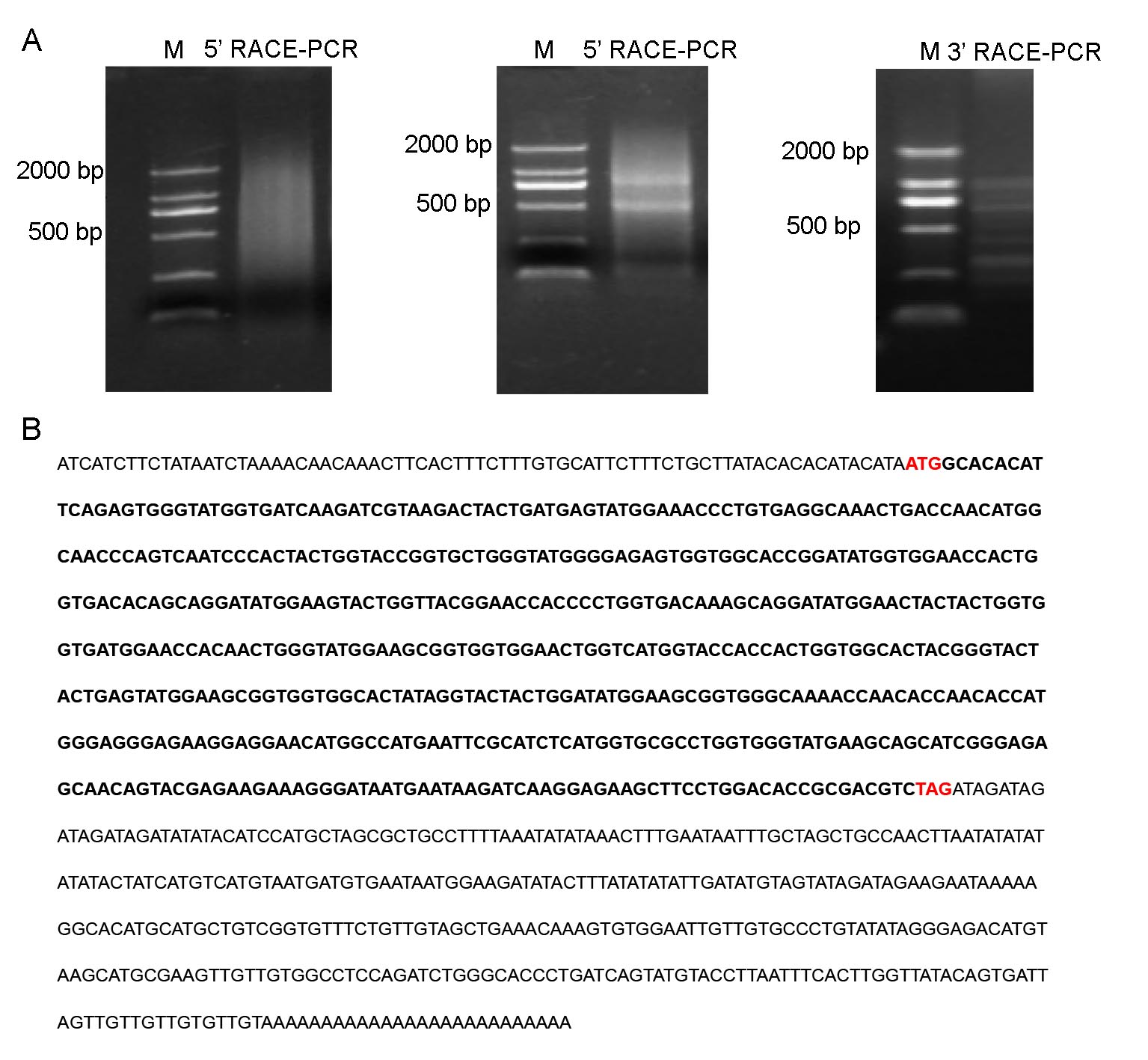

Supplement: Supplementary Figure 1 — The transcript of AnDHN. DHN, dehydrins. (A) Detection of the 5′ and 3′-terminus of the transcript of AnDHN by 5′ and 3′-RACE. The gene special primers were given in Supplementary Table 4 for detailed information. (B) The complete transcript of c195333_g1_i1 was obtained by 5′ and 3′ RACE. The start and stop codons were labeled in red. DHN, dehydrins; RACE, rapid amplification of cDNA ends. The bold sequence represents the coding sequence of AnDHN. [file Image_1.JPEG]

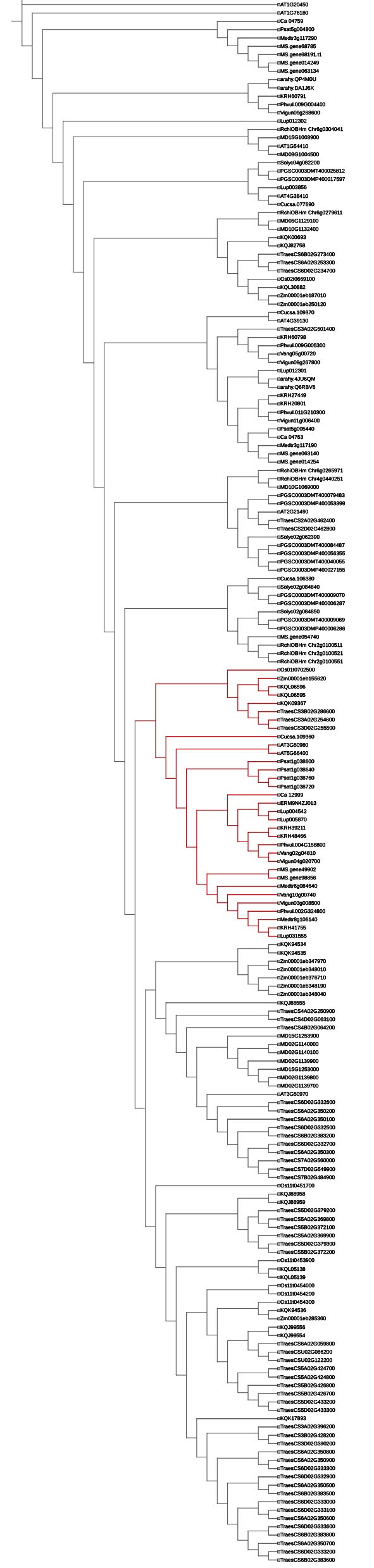

Supplement: Supplementary Figure 2 — A maximum-likelihood phylogenetic tree of DHNs. The generated dataset consists of 23 species and 189 DHN proteins. DHN, dehydrins. [file Image_2.JPEG]

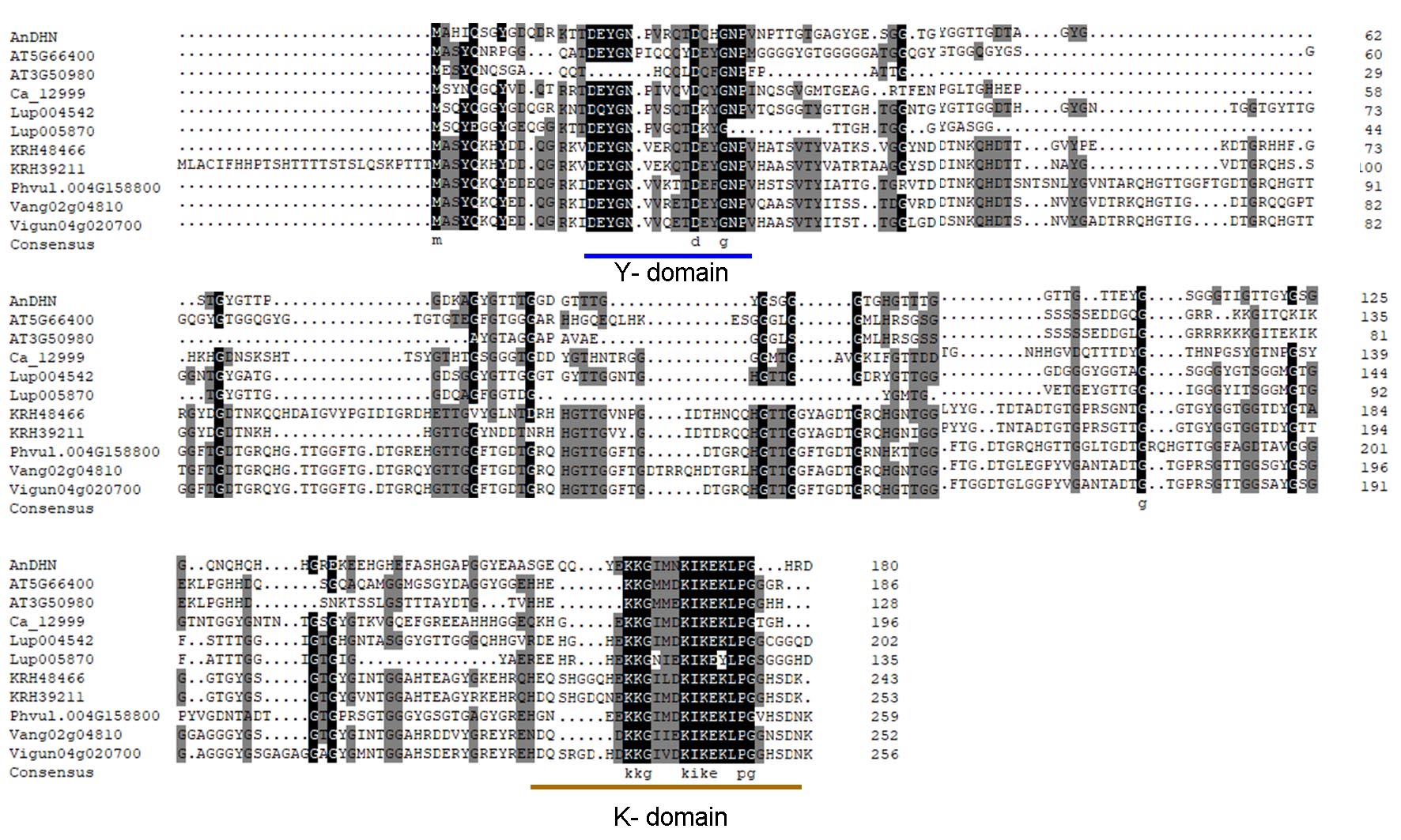

Supplement: Supplementary Figure 3 — Amino acid sequence alignments with closely related DHNs. DHNs from A. nanus and closely related species, namely, Arabidopsis, C. arietinum, L. angustifolius, G. max, P. vulgaris, V. angularis, and V. unguiculata. The Y-domain and K-domain were underlined in blue and brown lines, respectively. DHN, dehydrins. The black and gray highlights represent the conservative interval of the protein of AnDHN. The black highlight represents the sequence identified more than 75% and the gray highlight represents the sequence identified more than 50%. [file Image_3.JPEG]

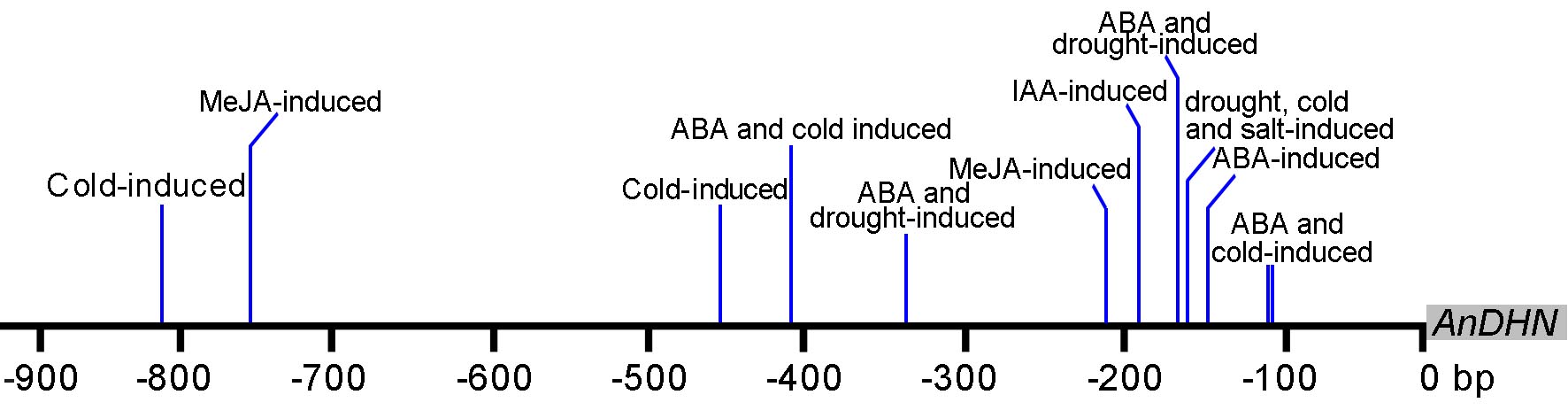

Supplement: Supplementary Figure 4 — Predicted cis-regulatory-elements (CREs) in the 830 bp promoter regions of AnDHN. DHN, dehydrins. [file Image_4.JPEG]

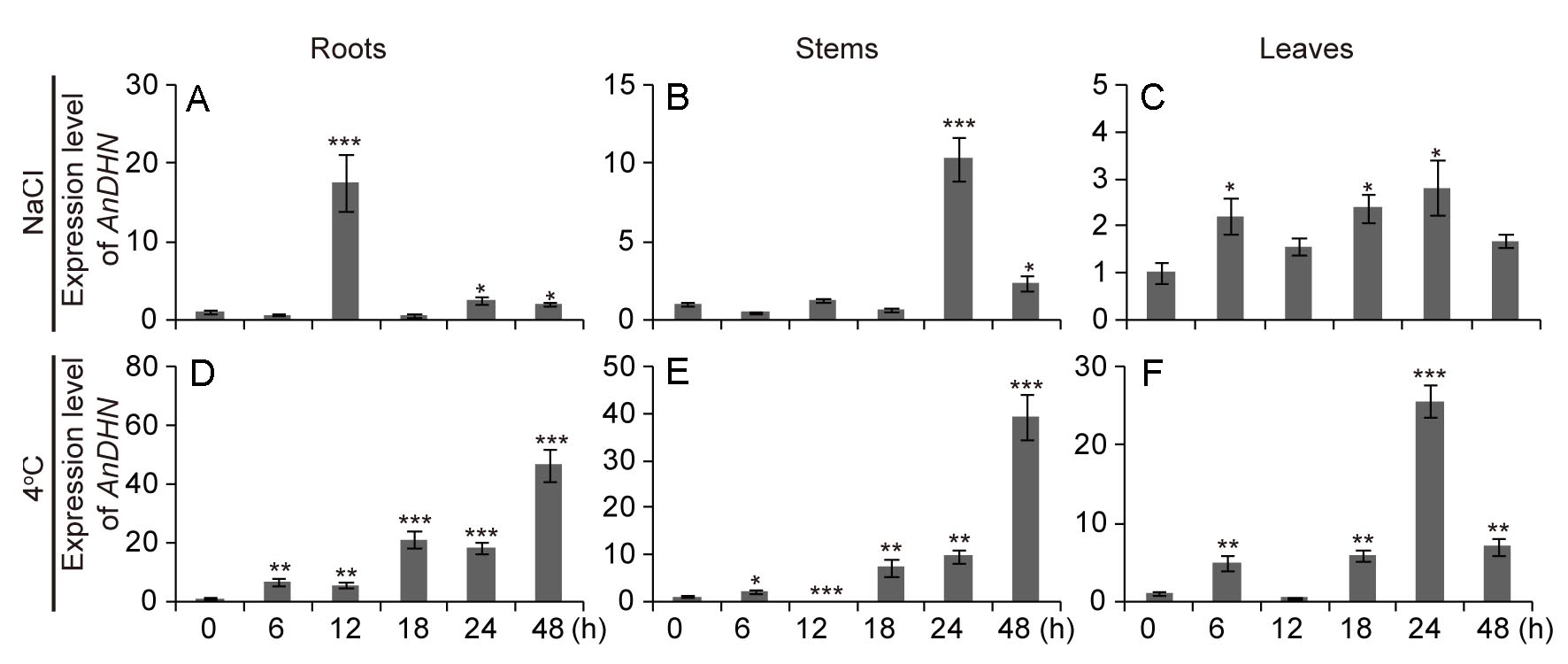

Supplement: Supplementary Figure 5 — (A–C) Quantitative RT-qPCR analysis revealed AnDHN expression level in roots, stems and leaves in different times under150 mM NaCl treatments. (D–F) Quantitative RT-qPCR analysis revealed AnDHNexpression level in roots, stems and leaves in different times under 4°Ctreatments. AnACTIN was used as an internal control. AnDHN expression wascompared with that in 0 h, the value of which was set as 1. Significant variationwas estimated with Student fs t-test *, p < 0.05, **, p < 0.01, ***, p < 0.001. [file Image_5.JPEG]

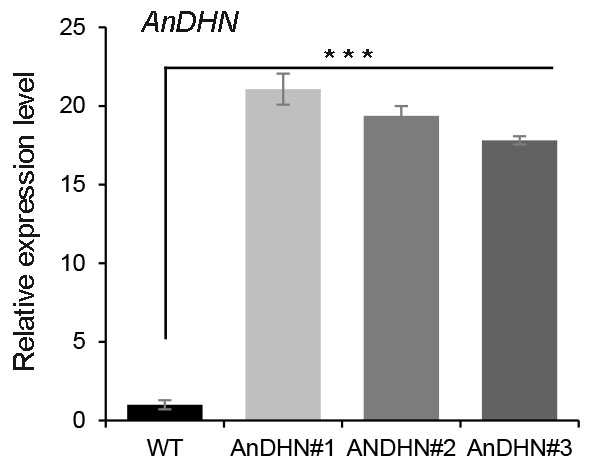

Supplement: Supplementary Figure 6 — Expression of AnDHN in the overexpression lines. RNA was isolated from 10-day-old seedlings. AtACTIN gene was used as an internal control. Significant variation was estimated with Student’s t-test of three independent biological replicates. ***, p < 0.001. [file Image_6.JPEG]

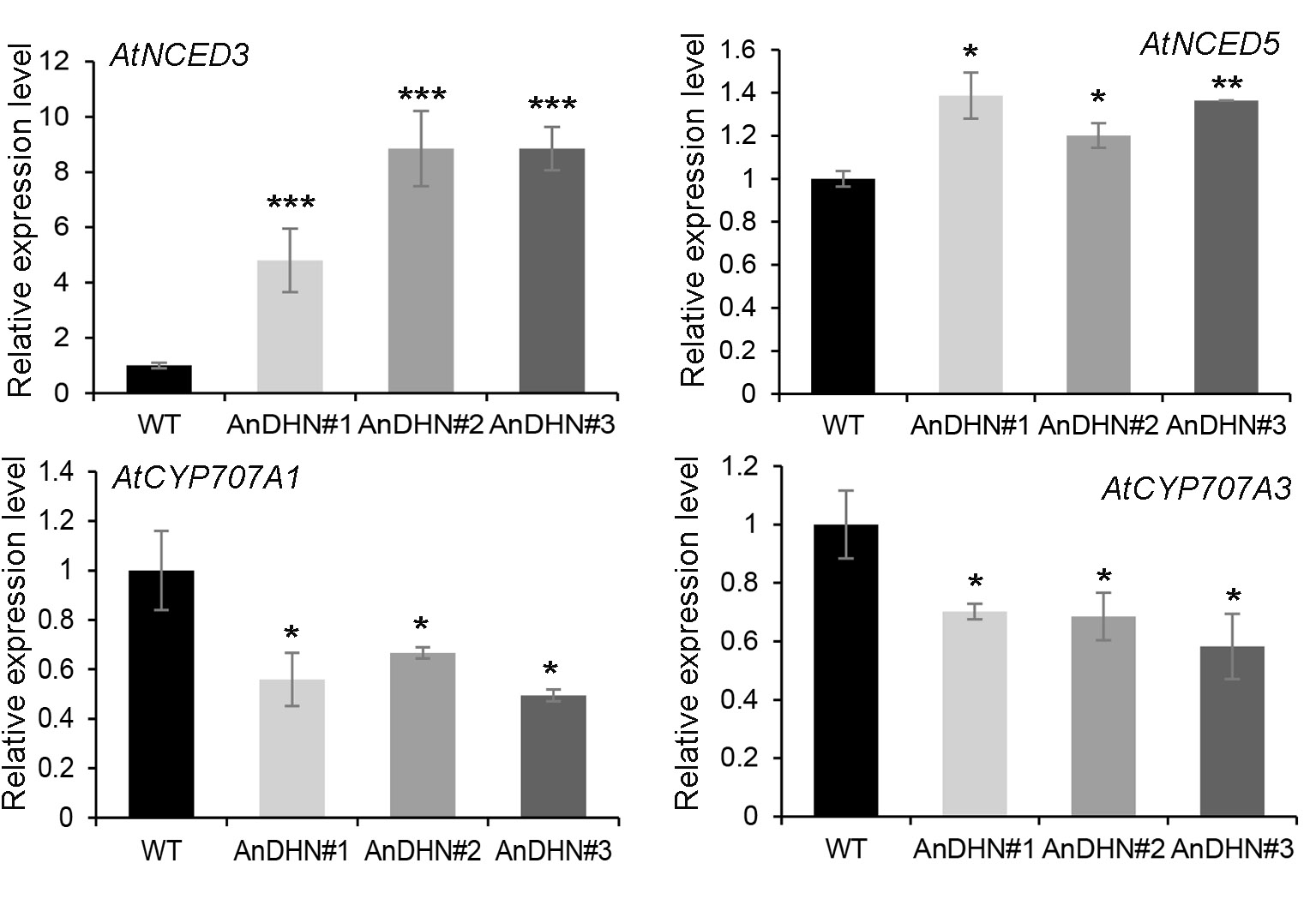

Supplement: Supplementary Figure 7 — The relative expression level of ABA biosynthetic and catabolic genes of WT and AnDHN overexpression lines. RNA was isolated from 10-day-old seedlings. AtACTIN gene was used as an internal control. Significant variation was estimated with the Student’s t-test of three independent biological replicates. *p < 0.05, **p < 0.01, ***p < 0.001. WT, wild-type; DHN, dehydrins. [file Image_7.JPEG]

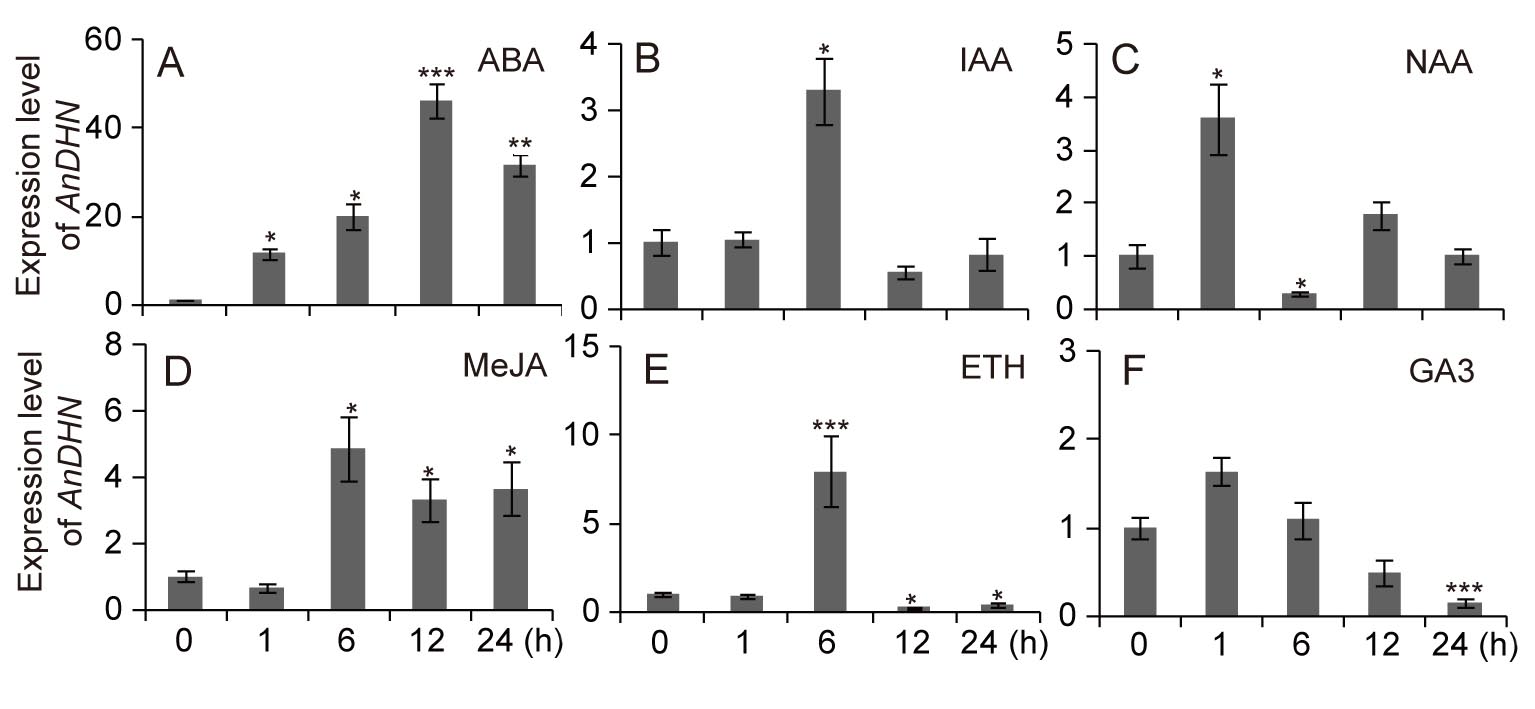

Supplement: Supplementary Figure 8 — Quantitative RT-qPCR analysis revealed AnDHN expression level in leaves in different times. (A–F) Were used ABA, NAA, IAA, MeJA, ETH, GA3 treatment, respectively. AnACTIN was used as an internal control. AnDHN expression was compared with that in 0 h, the value of which was set as 1. Significant variation was estimated with Student’s t-test, *, p < 0.05, **, p < 0.01, ***, p < 0.001. [file Image_8.JPEG]
